# Atrial Fibrillation: Mechanisms and Management

**DOI:** 10.1155/2019/8909371

**Published:** 2019-07-11

**Authors:** Tong Liu, Gary Tse, Lilei Yu, Panagiotis Korantzopoulos, Konstantinos P. Letsas

**Affiliations:** ^1^Tianjin Key Laboratory of Ionic-Molecular Function of Cardiovascular Disease, Department of Cardiology, Tianjin Institute of Cardiology, Second Hospital of Tianjin Medical University, Tianjin 300211, China; ^2^Department of Medicine and Therapeutics, Faculty of Medicine, Chinese University of Hong Kong, Hong Kong, SAR, China; ^3^Li Ka Shing Institute of Health Sciences, Faculty of Medicine, Chinese University of Hong Kong, Hong Kong, SAR, China; ^4^Department of Cardiology, Renmin Hospital of Wuhan University, Wuhan, China; ^5^Cardiovascular Research Institute, Wuhan University, Wuhan, China; ^6^Hubei Key Laboratory of Cardiology, Wuhan, China; ^7^First Department of Cardiology, University of Ioannina Medical School, Ioannina, Greece; ^8^Second Department of Cardiology, Laboratory of Cardiac Electrophysiology, Evangelismos General Hospital of Athens, Athens, Greece

Atrial fibrillation (AF) is the most common cardiac arrhythmia encountered in clinical practice, contributing to significantly mortality, and morbidity through the development of stroke, heart failure, and dementia. It has a prevalence of around 2% in the general population globally, but this is expected to increase exponentially in part owing to an ageing population. The current treatment modalities include pharmacological therapy and interventional procedures such as catheter ablation. However, both modalities have their shortfalls, which may be due to the fact that their pathophysiological mechanisms are incompletely elucidated. In this special issue of *Cardiology Research and Practice*, we have selected the latest original and review articles on the mechanisms and management of AF for our readers.

Regarding the risk factors and epidemiology of AF, this has been the topic of intense investigations. In addition to traditional risk factors such as diabetes mellitus, the relationship of malignancy and AF is complex. AF development is dependent on not only the cancer type, burden, and metabolic links but also different cancer therapies. The Xia group has conducted a detailed systematic review and meta-analysis, demonstrating that the risk of AF to be highest within 3 months of a cancer diagnosis. This has important implications for the area of cardio-oncology practice, where cancer patients may benefit from early intensive monitoring for the presence of AF and treatment for stroke prevention. The Fedele group provided a review on the possible pathophysiological mechanisms through which anxiety disorders can promote the onset, progression, and maintenance of AF. This highlights the importance of emotional stress as the source of triggers for this arrhythmia.

For novel biomarkers, the Zhang group conducted a prospective cohort study of patients with persistent AF and identified anti-M2-muscarinic acetylcholine receptor antibodies to be a biomarker for atrial fibrosis severity in the left atrial appendage, and this was associated with upregulation of transforming growth factor-*β*1 and connective tissue growth factor, which may represent future pharmacological targets. Novel biomarkers may not be readily available in all centers, and thus there has been investigation into the roles of routine markers from routine blood tests for risk stratification. In this issue, the Su group examined one of these markers, red cell distribution width, and found it to be an independent risk factor of AF and affected by the type of AF and altitude.

In terms of advances in pharmacological therapy for AF, the Liu group conducted a randomized controlled trial to investigate whether the traditional Chinese medicine, Wenxin Keli, provided additional benefits when added to usual amiodarone treatment. They found that the use of Wenxin Keli was safe and led to reduction in conversion time to sinus rhythm in patients with recent onset AF.

Catheter ablation is an invasive strategy used for the treatment of AF. Although it can be effective, recurrence can occur in a subset of patients, which can contribute to the costs and morbidity through the need of repeated procedures and the development of procedural complications. Therefore, there is a need to improve long-term success by identifying technical and mechanistic factors that can be used by clinicians to reduce arrhythmia recurrence. The Zhou and Ding group has conducted a pilot study to investigate the clinical utility of an automated lesion tagging module based on catheter stability information, VisiTag, with the CARTO system. They found the optimal predefined criteria (OPC) to be 3 mm distance limit for at least 20 seconds and Force Over Time of 5g in pulmonary vein isolation procedures. The Wang group has identified concealed bigeminy arising from the pulmonary veins during sinus rhythm as a marker of pulmonary vein firing. This can guide interventionalists to induce a greater number of radiofrequency ablations to improve isolation. Previously, pharmacological challenge tests such as adenosine testing to unmask dormant conduction failed to demonstrate a significant benefit on long-term AF recurrence rates. Concealed pulmonary vein potentials may be unmasked electrically by left atrial stimulation. Nevertheless, the Liu group explored the use of low-dose ibutilide, a Class III anti-arrhythmic agent, during catheter ablation in persistent AF patients and found that it can be used for substrate localization and possibly reduction of ablation of unnecessary sites. To prevent stroke, left atrial appendage occlusion is a well-recognized treatment and is typically guided by a combination of transoesophageal echocardiographic imaging and fluoroscopy. The Zhang group investigated a new method of left atrial appendage occlusion using transoesophageal echocardiographic imaging alone without fluoroscopic guidance. In their article, they reported the feasibility and safety of this novel approach, with implications that the procedure can be simplified and radiation risk can be reduced to healthcare workers and patients underlying this procedure. Finally, the Mei group investigated the use of catheter-based renal denervation procedures to reduce cardiac sympathetic nerve activity, hypothesizing that this can inhibit AF in a canine model. They found that renal artery adventitial cryoablation could create transmural effects by applying lesions from outside of the renal artery, leading to neural modelling, as well as decreases in AF incidence and duration. Using a rabbit model, the Huang group investigated the pathophysiology of exercise training-induced AF and identified downregulation of two repolarizing currents, I_K1_ and I_KACh_, as the underlying atrial remodelling mechanisms.

In conclusion, this special issue published clinical and experimental studies on recent advances regarding the epidemiology, mechanisms and pathophysiology, biomarkers, and pharmacological and nonpharmacological management of AF.

